# Is Human T Cell Lymphotropic Type 1 (HTLV-1)-Associated Myelopathy/Tropical Spastic Paraparesis (HAM/TSP) Syndrome a Neglected Disease?

**DOI:** 10.1371/journal.pntd.0000487

**Published:** 2009-11-24

**Authors:** Jorge Casseb

**Affiliations:** Laboratory of Medical Investigation LIM-56/Faculty of Medicine-USP, Institute of Tropical Medicine of São Paulo–University of São Paulo, São Paulo, Brazil; London School of Hygiene & Tropical Medicine, United Kingdom

## Introduction

Human T cell lymphotropic type 1 (HTLV-1) was the first human retrovirus discovered and has been associated mainly with two illnesses [1], an inflammatory disease named HTLV-1-associated myelopathy/tropical spastic paraparesis (HAM/TSP), and a neoplastic condition called adult T cell leukemia/lymphoma (ATL) [2]. Although this virus has worldwide distribution, Japan, Africa, the Caribbean basin, and South America are considered to be endemic areas [3]. It has been estimated that 10–20 million people are infected with this virus [4], but the majority are asymptomatic and probably not aware of their serological status. There is no accurate number of HAM/TSP or ATL cases since these diseases are not considered reportable by the World Health Organization (WHO), although Japan reports approximately 800 cases of ATL yearly [5]. In 1985, Roman et al. used the term tropical spastic paraparesis (TSP) for the first time, modifying the term “tropical spastic paraplegia” as used in South India in 1969, because only a few cases were completely paraplegic [6]. In the same year, it was found that almost 60% of patients with TSP were also positive for HTLV-1 compared with 4% of the controls, which suggested, for the first time, the neurotropism of human retroviruses [7]. In 1986, Osame et al. coined the term HTLV-1-associated myelopathy (HAM) [8]. In 1988, the WHO recommended “that the disease be known by the acronym HAM/TSP for the time being.” Thus, it seems that in endemic areas, about 60% of tropical spastic paraparesis cases are identified as HAM/TSP [7]. The rest are myelopathies probably caused by nutrition problems, intoxication, and unknown causes [6],[9].

HAM/TSP has an estimated incidence ranging from 0.25% to 1% after 30–40 years of incubation [9]. The onset of disease is 40 years of age, with predominance in women [10]. Several factors have been ascribed as potentials for clinical outcome, such as high HTLV-1 proviral load, genetic background, routes of transmission (i.e., breastfeeding or transfusion), and high antibody titers [11].

Despite the publication of several reviews regarding the pathogenesis or molecular biology of HTLV-1 [12],[13], few studies have addressed treatment for the diseases caused by this virus. Thus, this article will focus on the reason why HAM/TSP should be considered a neglected tropical disease.

To illustrate our viewpoint, we present one case of HAM/TSP in which several important issues are raised as singularities of the problem. A 29-year-old black woman born in Bahia in northeast Brazil has been living in São Paulo city for several years. When she was 20, she began complaining of lumbar pain and parestesis, initially in one leg and then in both, in addition to miccional urgency and constipation. After 3 years of illness and several visits to doctors, including basic and intermediate complexity level services, she was referred to our service as a suspected case of HTLV-1 disease. The diagnosis of HAM/TSP was confirmed. The patient was using a wheelchair most of the time. Pulse therapy with methylprednisolone was administered three to four times per year, with programmed hospitalization for at least 5 days. Her husband abandoned her, and she lives with her two children in a small one-bedroom house in an area difficult to reach by car. Her only income is the government minimum wage (US$250.00/month), and she is unable to attend a facility for physical therapy. She depends on her friends or relatives to bring her to the clinic appointments.

## There Is No Specific International Classification of Disease for this Condition

The neurological disease TSP/HAM has no International Classification of Disease (ICD-10) code. In our clinical practice, we use the G04.1 code to designate this condition. This code means Tropical Spastic Paraplegia. In fact, many of our patients, when they need social security assistance, must present one report of ICD-10 by an attending physician. For this reason, we provide an ICD-10 closest to the clinical features of HAM/TSP. The creation of an ICD-10 code specifically for HAM/TSP would solve this problem and avoid any complications for physicians when they provide this report. Furthermore, this specific and more accurate code would also allow for the surveillance of the number of cases in the population, if this condition becomes an obligatory reportable condition by WHO in the future.

## Neurologists and Orthopedists, as Well as Other Health Professionals, Have Little or No Knowledge of This Condition, and This May Have Great Impact on the Accuracy of Diagnosis

We believe that the main reason for the low level of knowledge about HAM/TSP is the difficulty of diagnosis. The current guideline was recently updated by experts from several parts of the world [14]. It is possible that recommendations are too restrictive and specific. Easier recommendations should be implemented to facilitate diagnosis and reporting by non-HTLV experts in the clinical setting, especially in developing countries.

Based on our clinical experience here in Brazil, we suggest that HAM/TSP be characterized as a chronic, slowly progressive, spastic paraparesis with bladder disturbances, absent or mild sensory loss and low back pain, and positivity for HTLV-1 antibodies in serum and cerebrospinal fluid [7]. The HAM/TSP diagnosis must exclude spinal cord compression, hypovitaminosis of B complex, hypo- or hyperthyroidism, or other causes of pyramidal syndrome, and impotence in men is a common picture. Overall, one-fifth of patients may experience a rapid progression, with severe disability two years after the onset of symptoms [15].

Because of the symptoms and signs of HAM/TSP, most patients are referred to or are seen for the first time by neurologists, orthopedists, urologists, or other health professionals involved in the rehabilitation process (physical therapists, for example). These professionals may have little knowledge of HAM/TSP or HTLV-1 infection, and a lack of information regarding this condition may delay the diagnosis. In fact, the mean time for correct diagnosis of HAM/TSP patients in our clinic was 7 years. Carod-Artal et al. found that 17% of the patients who were followed up in the most important rehabilitation hospital in central Brazil (in Brasilia, Brazil's capital) had HAM/TSP diagnosis, with a mean time of diagnosis of 11 years [16]. It is an important issue since early recognition of the disease may improve the prognosis and the response to treatment [17].

This problem could be addressed by improving the teaching of HTLV during undergraduate training and medical residency in places where HTLV-1 is endemic. In addition, lectures and/or case discussions during national meetings of these specialties could provide better information for these professionals. Furthermore, guidelines such as those released by the US Centers for Disease Control as well as Brazilian HTLV guidelines should be updated and be universally accepted [18],[19].

One good strategy for providing better information to the public as well as to health care providers is using the Internet. A Web site was created in 2003 with general information regarding HTLV infection and the associated diseases, as well as information for the general public in non-technical terms to address the most frequently asked questions (http://www.htlv.com.br). This Web site has information in three languages, Portuguese, Spanish, and English, and for the first year had financial support from the Ministry of Health of Brazil. There is considerable interest in this site, with an average of 5,000 visits per month.

## No Useful Treatments Are Available

Despite the 24 years that have passed since the first HAM/TSP cases were described in Martinique, no successful treatment is available to treat HAM/TSP patients (reviewed by [20]). Most treatments have been directed at reducing inflammation in the affected tissues. The use of interferon-α, oral prednisolone, intrathecal hydrocortisone, plasmapheresis, vitamin C, and antiretroviral drugs has been reported. There are some reports of transient benefits of immunotherapy, pentoxyfiline, danazol, milk drinks containing *Lactobacillus casei*, and more recently, valproic acid [21]. Even though corticosteroids are the most widely used therapy for TSP/HAM, few clinical trials with corticosteroids have been published recently. In our experience, only 25% of patients showed neurological improvement with the use of corticosteroids, with physical therapy and anti-spastic drugs as adjunctive treatment [22]. Thus, larger multicenter randomized clinical trials to assess the use of corticosteroids and other potentially useful immune-based therapies for HAM/TSP treatment are urgently necessary.

Finally, recognition of HAM/TSP as a neglected disease would provide more focus on this condition, perhaps increasing the number of researchers in this field, which could lead to the development of better treatment options for patients and also avoid the confusion between HTLV-1 and HIV-1 (even at a professional level) without adding further stigma on these individuals.
